# Applying mobile eye tracking to measure real-time engagement and enhance informal learning at environmental exhibits

**DOI:** 10.3389/fpsyg.2025.1607029

**Published:** 2025-12-09

**Authors:** Monika Lohani, Lynne Zummo, Alec G. Roberts, Ginger R. Blodgett

**Affiliations:** 1 Department of Psychology, University of Utah, Salt Lake City, UT, United States; 2 Natural History Museum of Utah, University of Utah, Salt Lake City, UT, United States; 3 Department of Educational Psychology, University of Utah, Salt Lake City, UT, Unites States

**Keywords:** informal learning, visual engagement, applied environmental research, innovation, climate change, mobile eye tracking methods

## Abstract

Mobile eye-tracking is a valuable method that holds significant potential for understanding informal learning in applied environmental science settings, yet it remains underutilized. In this paper, we explore the benefits of adopting this technology to objectively assess engagement in real-time, which can inform the design and advancement of informal learning through environmental science exhibits. In addition, mobile eye-tracking offers technology to conduct state-of-the-art research in applied museum settings. Using a climate change exhibits as an example, we illustrate ways to leverage this technology to improve exhibit design and deepen our understanding of visual engagement and informal learning. Finally, we address the challenges and areas for growth in applied eye-tracking technology. Overall, we present what this cutting-edge methodology can offer to enhance engagement and learning through environmental exhibits.

## 1 Introduction

Exhibits are reliable ways to curate and conserve culture, diversity, and scientific knowledge (American Alliance of Museums, 2018). They provide an open and informal learning environment that can spark new ideas and bring communities together. An important goal of creating exhibits is to present information that is objective, engaging, and thought-provoking (American Alliance of Museums, 2018), and, in doing so, can promote knowledge gain and interest in science among learners (e.g., Martin et al., 2016). However, the degree to which exhibits promote visitor engagement can vary across different types of exhibits (Shaby et al., 2017). The level of engagement with exhibits by visitors influences the learning that occurs through an exhibit, as engagement is related to the meaning-making experienced by visitors (e.g., Barriault and Pearson, 2010; DeWitt et al., 2019). Thus, it is critical to understand which aspects of an exhibit facilitate deep engagement and which do not. Prior methods used to understand engagement typically include *post-hoc* surveys, interviews, or observation, with new methods in video analysis also emerging (Mujtaba et al., 2018). In addition to these more commonly utilized approaches, it would also be valuable to learn about visual engagement in exhibits more objectively by tracking visitors in real-time, which remains extremely limited.

The aim of the current work is to highlight the application of mobile eye tracking as a state-of-the-art methodology to improve exhibit design and conduct empirical research of visual behavior in museum settings. This paper combines existing theoretical and empirical perspectives to demonstrate the relevance and benefits of adopting eye tracking methodology in museum exhibits. To highlight the methodological approach, we present pilot data collected at a climate change exhibit to showcase the qualitative and quantitative approaches that can be applied to museum research. Even if the exhibit topic may not match the readers' area of work, it will still be useful to utilize a specific exhibit with data to discuss examples of qualitative and quantitative interpretations and decisions that can be made through eye movement data. In terms of layout, we first introduce the use and application of mobile eye tracking methodology in assembling and investigating exhibits that are engaging and conducive to meaningful learning. Next, we provide a brief overview of the eye tracking methodology, followed by its potential benefits and applications. We draw on the example of a climate change exhibit to illustrate how this technology can be applied in practice. Finally, we discuss considerations for adopting this technology to promote learning outcomes through environmental exhibits. Overall, this paper aims to highlight the merits of adopting mobile eye-tracking as an innovative and cutting-edge approach to improving learning through exhibits on climate change and sustainability.

**Figure 1:**
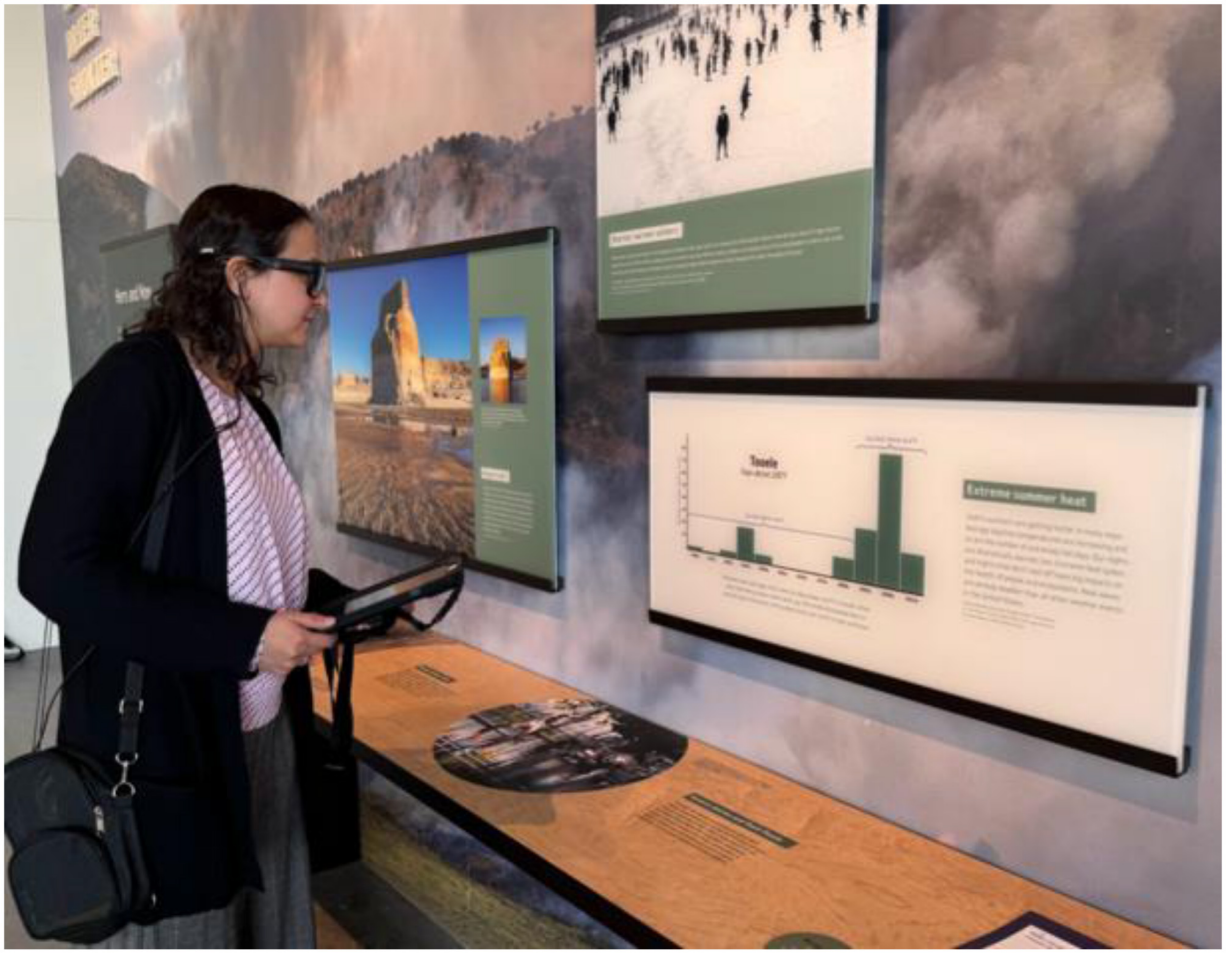
A visitor exploring a climate change exhibit while wearing a mobile eye tracker. Additionally, a tablet (held in hand) can be integrated to answer questions (e.g., experiences) and complete tasks (e.g., discuss with others) in real-time.

**Figure 2:**
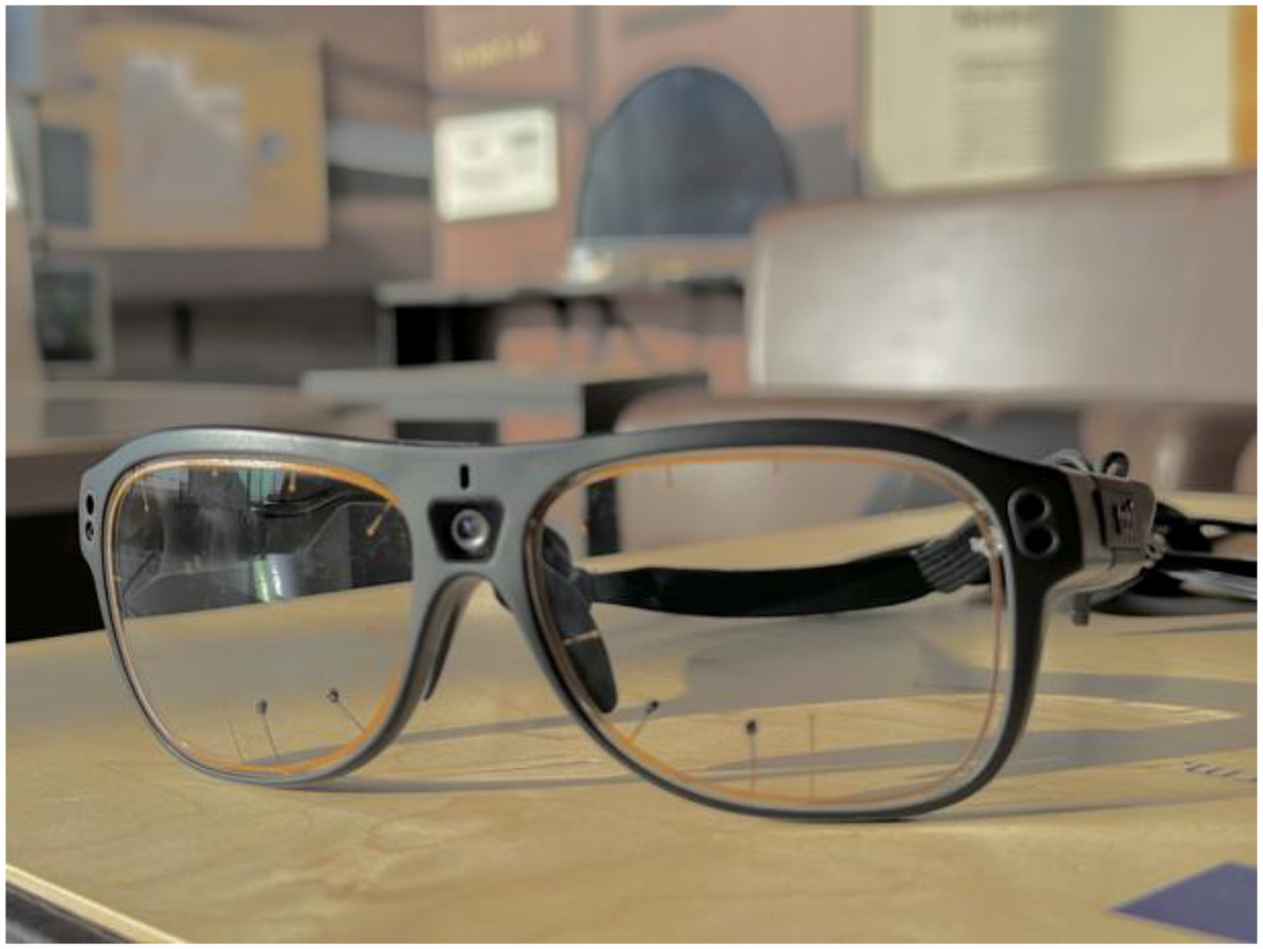
Example of a mobile eye-tracker (Tobii Pro Glasses 3) with its illuminators and cameras.

**Figure 3:**
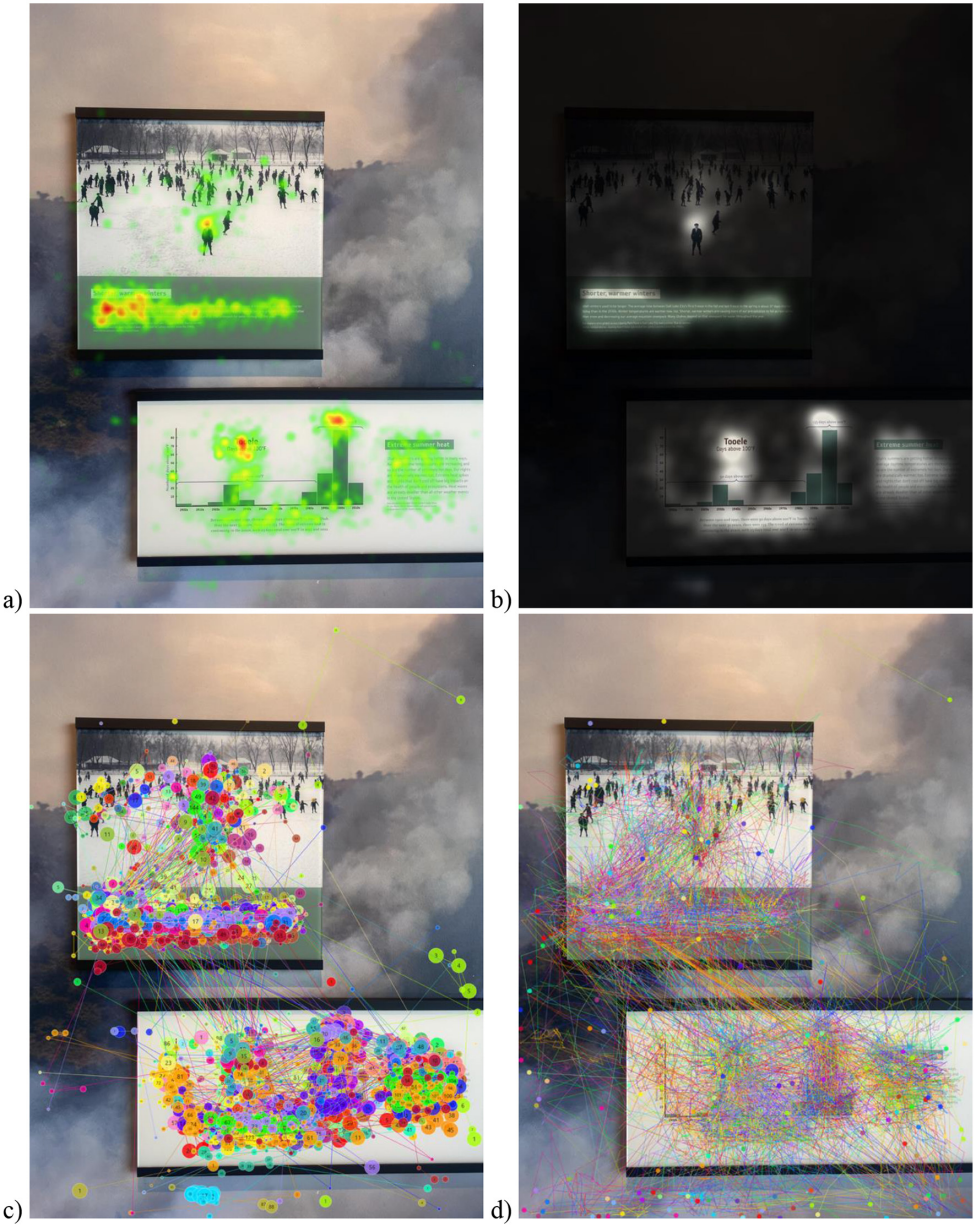
Examples of different data visualizations are presented (based on data from 50 participants). The same portion of the exhibit is presented with four different types of visualizations: **(a)** Heat maps, **(b)** Opacity maps, **(c)** Scan path, and **(d)** Beeswarm.

**Figure 4:**
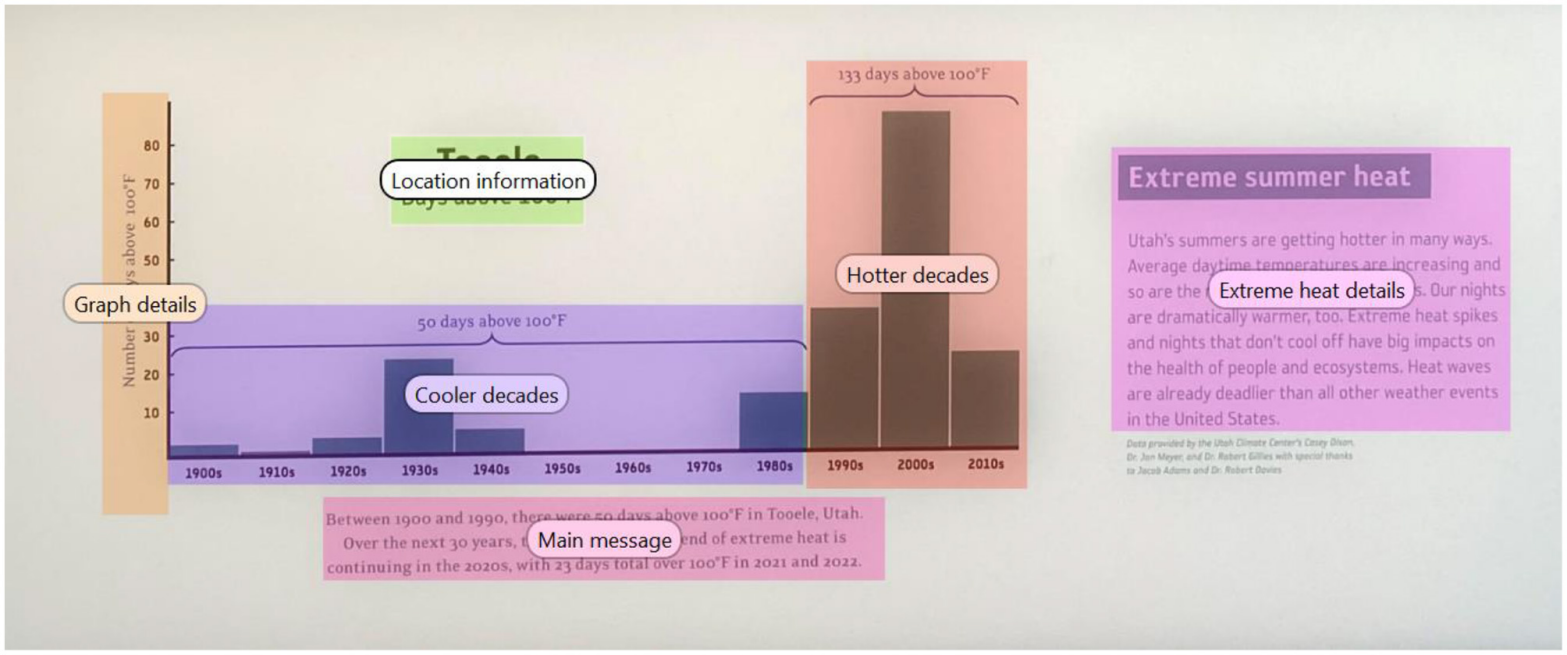
A portion of a climate change exhibit that presents manually created and superimposed Areas of Interest (in the form of colored objects with labels) that are then used for data visualizations and metrics generation purposes.

The *eye tracking methodology* monitors and records the eye positioning and movements by utilizing sensors and cameras. The *gaze* is where a person is visually looking, and it is the focus of eye tracking technology (see Figure 1). The *eye tracker* is equipped with sensors that allow the researcher to detect and measure the visual gaze and scanning patterns of its wearer (see Figure 2). This information can be detected in real-time and also stored for further examination post-processing to generate visualizations and indices to learn where, how long, and in what order the wearer looked at the presented content. Visualizations are qualitative approaches (see Figures 3, 4). Quantitative information on gaze behavior can be monitored in two common ways—fixations and saccades (Rayner, 2009). A *fixation* is a time period when the eyes gaze at an area and remain there for a minimum duration, usually representing visual engagement (e.g., Manor and Gordon, 2003; Rayner, 2009). *Saccades* are swift eye movements between fixations and are often associated with search patterns (Phillips and Edelman, 2008; Rayner, 2009). More details are further discussed in the section, Technical information on mobile eye tracking. The eye-tracking methodology has a long-rooted history that has spanned over a century and can be used to understand visual gaze patterns across wide-reaching domains (Bell and Davy, 1823; Dodge and Cline, 1901; Płużyczka, 2018). Eye trackers have continually grown in complexity and accuracy since this point and now have applications across a broad set of domains (Liversedge et al., 2011), such as consumer research, driving, medicine, and education (Harwood and Jones, 2014; Kapitaniak et al., 2015; Ke et al., 2024; Kwon and Kim, 2021; Liu and Cui, 2025; Pavisic et al., 2017; Sharon, 2017; Tóthová and Rusek, 2025).

Eye-tracking research is grounded in principles of cognitive psychology. Interpretations about mental processes or the stimuli of interest have long rested on classic premises in the field that individuals begin processing information as soon as it enters their visual field, known as the *immediacy-of-processing assumption*, and that they maintain their fixation until processing is complete, called the *eye-mind assumption* (Just and Carpenter, 1976). These assumptions continue to guide modern interpretations of eye-movement data being linked to cognitive processing. At the same time, subsequent research has revealed important nuances and exceptions, especially in more naturalistic contexts (Kuperman et al., 2013). For example, attention can sometimes be disengaged from eye movement, such as when it is directed internally (Henderson, 2003). Still, the hypothesis that eye movements are associated with cognitive processes, along with extensive empirical research, has helped make meaningful interpretations about the direction of the viewer's attention through their eye movements (e.g., Duchowski, 2017; Just and Carpenter, 1976; Hessels et al., 2025; Hooge et al., 2025; Rayner, 2009, 1998). Therefore, attention is often aligned with the location of the viewer's gaze while interacting with the external environment. As a result, eye-tracking remains a highly valuable tool for studying attention to both static and natural scenes (Henderson, 2003). In the museum context, eye tracking data offers an accurate and non-invasive way to measure concepts relevant to exhibit design, engagement, and learning.

On a related note, the constructivist theory of learning (Piaget, 1954) suggests that knowledge is gained by actively interacting with one's environment. More recently, Pande (2021) has synthesized how individuals may integrate information from the external environment and the internal representations, specifically in a STEM learning context. The *field theory model of external representation integration* (Pande, 2021) proposes how a learner's mind may integrate information from their external environment (e.g., museum exhibits) and internal representations (e.g., perception, attention) through sensory processing and action in order to learn from their environment. This theory extends the understanding of integration of external and internal representations (Hegarty, 2004; Prinz, 1997) and allows for a deeper exploration of individualistic processes that may vary across individuals (Menary, 2007). The naturalistic environment available at an exhibit can be extremely conducive to testing existing cognitive and learning theories while visitors engage in the real-world context of a museum. Relatedly, the use of the mobile eye tracking methodology enables a systematic examination of the perceptual, attentional, and learning processes that occur while museum visitors interact with exhibits. To further explicate its relevance, we discuss some applications of mobile eye tracking to museum exhibits.

### 1.1 Application of mobile eye-tracking to informal learning at exhibits

#### 1.1.1 Exhibit design and improvement through theoretical and empirical perspectives

Museum exhibits hold great potential as places of learning (e.g., Falk and Dierking, 2000; Rainoldi et al., 2018). From a theoretical perspective, unfacilitated exhibits offer opportunities for constructivist learning, where learners build their own understanding by integrating new ideas from the exhibit with their existing cognitive resources (e.g., Allen, 2004; Bransford et al., 2000). Additionally, when accompanied by friends, family, or significant others, exhibits offer social opportunities in which learners can come to understand new ideas through engagement with both the exhibit and their companions (e.g., Ash, 2003; Blud, 1990; Oler et al., in press). However, learning through exhibits, given that such learning is self-directed, relies on substantial engagement with exhibits (Allen, 2004), which can vary across types of exhibits and learners (e.g., Shaby et al., 2017). Specifically in the context of museum exhibits, there has also been a growth in technologically advanced and interactive displays. A precursor to learners' constructing new knowledge within museum exhibits, then, relies on prolonged engagement. To afford visitors learning opportunities through exhibits, museums have the difficult task of presenting scientifically or historically accurate information to a diverse audience, many of whom spend only brief moments engaging with any given exhibit. Consequently, museums must optimize exhibit design to effectively capture visitors' attention and convey key information (Allen, 2004; Garbutt et al., 2020; Mayr et al., 2009). In Figure 1, we show a museum visitor going through an exhibit while wearing a mobile eye tracker. The mobile eye-tracking technology can be quite useful at various stages of exhibit development and maintenance.

During the *initial stages of exhibit* development, pilot data can provide useful information in making decisions about exhibit design. Eye trackers can indicate where and for how long museum visitors are engaging with particular sections of exhibits. Existing research on scene perception provides useful knowledge about where and how long visitors look at a section of an exhibit is linked to visual attention (Kümmerer and Bethge, 2023; Nuthmann, 2017; Nuthmann and Canas-Bajo, 2022; Williams and Castelhano, 2019). During the piloting process, this information can be valuable in ensuring that the visual behavior of visitors aligns with the plans for the exhibit. Similarly, initial eye tracking data can be collected to identify portions of the exhibit that are hard to follow or are less engaging to visitors and can be improved before the exhibit is opened to the public. This information can be tremendously useful in streamlining the finalists for the exhibit, as it can help to parse which aspects of an exhibit can foster high levels of engagement (e.g., Shaby et al., 2017). Hence, the mobile eye tracking methodology can enhance the exhibit development process that prioritizes intrinsically motivating exhibits that sustain interest and engagement among visitors, thereby supporting learning toward intended outcomes (e.g., Allen, 2004; Garbutt et al., 2020; Mayr et al., 2009).

Furthermore, mobile eye trackers can also be adopted to *improve the exhibit* over time to make sure additional information can be assimilated into the exhibit for continued learning. For example, applying this methodology to the climate change context, we adopted this technology to identify portions of the exhibit that were less visually engaging to museum visitors (Oler et al., in press). The exhibits team can rearrange their exhibit with some replacements or relocations and test if the engagement with key exhibit details continues to occur. This is supported by a study that examined the viewing behavior before and after the artwork was rearranged in its permanent collection at the Austrian Gallery Belvedere (Reitstätter et al., 2020). This effort demonstrated the benefits of mobile eye-tracking in examining differences in exhibit design (Reitstätter et al., 2020). Use of mobile eye tracking methodology allowed researchers to quantify the differences in the attention of individual pieces of artwork and the exhibit on the whole before and after a redesign.

Additionally, mobile guides are an increasingly common way to provide visitors with personalized content that aligns with their interests and motivations for visiting an exhibit. They increase the time visitors spend on each exhibit (Mokatren et al., 2016). Mobile guides can be improved through the implementation of eye-tracking to suggest what information should be supplemented to the user and when (Mokatren et al., 2016). For example, someone who is trying to deepen their understanding of climate change might spend more time reading signs, which could trigger a mobile guide to suggest other supplementary materials. In contrast, someone who is learning about climate change for the first time may spend more time looking at images and might benefit from a mobile guide that allows the viewer space to process the information and suggests helpful ways to regulate emotional reactions to climate change. Therefore, eye trackers can be used to better understand how most people are spending their time in an exhibit and what supplementary information might be useful in a mobile guide.

#### 1.1.2 Research progress toward informal learning

In addition to being helpful in setting up and improving exhibits, the eye tracking methodology can be used to inform research in applied settings (Fu et al., 2024; Hou et al., 2025; Silva et al., 2019). Eye-tracking can provide objective, real-time, and unobstructed ways of studying visual engagement and related informal learning that can continue outside formal learning (e.g., schools; Jarodzka et al., 2021). Depending on the research questions of interest, additional measures can be assessed to understand their connections to exhibit content. For instance, before, during, and after visitors experience the museum, more information can be collected around affective experiences (positive vs. negative), memory (e.g., learning outcomes), traits (e.g., personality), identity (e.g., political affiliations), attitudes and beliefs (e.g., “is climate change real?”), interest and motivation, etc. These data can collectively inform understanding of the factors linked to engagement with a particular exhibit. Instant and delayed questions can also be built into the study protocol to investigate how learning outcomes are connected to visual engagement and other individual difference variables relevant to the research questions.

On a related note, eye tracking data can be simultaneously collected with additional measures in real-time to gain a comprehensive understanding of museum visitors' experiences. For example, as shown in Figure 1, a visitor is also carrying a digital tablet in her hand and, when prompted, can report questions specific to the exhibit or personal experiences in response to the exhibit. Such an ecological momentary assessment approach can be utilized to gain insights into the diverse experiences of visitors Lohani and Blodgett, (2025). Furthermore, video and audio information is also collected in real-time and synced with fixation data. Building on this existing concept, additional measures can be synchronized with eye tracking data, including physiological measures (e.g., heart rate and skin conductance).

## 2 Material and equipment

In terms of transportability, two types of eye trackers are currently available: stationary (static) and mobile (ambulatory); for details on different types of eye trackers, see Nyström et al. (2025). Originally, stationary eye trackers were developed, and advancing technology has made the development of mobile eye trackers possible. *Stationary eye trackers* are usually mounted in front of the participants while they are interacting with visual content (e.g., presented via a monitor), thereby limiting movement in the area where they are sitting or standing. These have been widely used in studying visual attention to a screen, such as to research attention, emotion, language processing, and psychopathology (e.g., Lohani and Isaacowitz, 2014; Pavisic et al., 2017; Purcell et al., 2018; Rayner, 2009; Payne et al., 2022). However, stationary eye trackers generally lack the ability to capture a naturalistic view of the visitor experience. This is because stationary eye trackers are not designed to handle head movement in naturalistic settings (such as a visitor at an exhibit), making it infeasible for such applied settings. As such, *mobile eye trackers* allow their wearer to be unrestrained (i.e., not attached to a computer via a wired setup) and free to physically move around and explore a defined space of interest (such as a museum). This enables the study of naturalistic behavior in museum exhibits. Thus, mobile eye trackers are more realistic to adopt in the museum context. Indeed, limited recent work has shown that mobile eye trackers do have the potential to be adopted successfully within the museum context (Oler et al., in press; Dondi and Porta, 2023; Mokatren et al., 2016).

## 3 Method

### 3.1 How does a mobile eye-tracker work?

To counter the issue of naturalistic movement, mobile eye trackers utilize multiple illuminators and cameras (that are placed onto a device that resembles a pair of glasses), which help detect eye positioning and movement. The illuminators are assembled on the devices around the eyes to illuminate them reliably without being influenced by other sources of light in the environment. Typically, the illuminators have near-infrared light (which is not visible to the human eye and is thus not distracting or uncomfortable), thereby creating reflections on the pupil (the inner circular center of the eye that allows light to enter the back of the eye) and the cornea (the clear dome-shaped protective surface of the eye). These eye reflections are captured by camera sensors, and further image processing software is utilized to estimate where the wearer is looking (i.e., their gaze). Overall, the relative distance between the reflection on the cornea and the center of the pupil is utilized to calculate the point of gaze. Thus, the information collected from different cameras can be combined to track the wearer's gaze with a lot of precision. Video-based eye trackers are commonly adopted and utilize a reflection in the cornea of infrared light in relation to the pupil (Carter and Luke, 2020).

For example, a recent mobile eye tracker (Tobii Pro Glasses 3, 2020; as shown in Figure 2) has five cameras per set of glasses. Four of these are used to track the movements of the eye and are located on the outer rim of the glasses, with two cameras dedicated to each eye. The fifth camera is located above the bridge and is used to record “scene video,” which is a regular recording from the wearer's vantage point as they move throughout the exhibit. Additionally, this model has a microphone that records audio (Tobii Pro Glasses 3, 2020, 2024), which can be utilized for additional analysis. While collecting data from this eye tracker, the glasses are worn by a wearer while exploring the exhibit, along with additional equipment (such as a tablet, to collect survey or response data in real-time, as shown in Figure 1). The glasses are remotely (wirelessly) controlled via a computer that allows researchers to start and stop data collection and provides a live feed from the tracker. The data from the cameras and microphone is then merged into a single video file that contains the wearer's view of the environment and their gaze. This allows researchers to virtually ride along with the wearers and see exactly what they see, yet not be in the physical vicinity of the wearer (which is relatively less disruptive than being in physical proximity). Furthermore, the accuracy of mobile eye tracking has been improving over subsequent generations (Onkhar et al., 2024). Therefore, newer models of eye trackers are more robust to changing conditions compared to earlier models, making naturalistic data collection at exhibits more feasible.

### 3.2 Pilot study details: application of mobile eye-tracking to a climate change exhibit

To make these discussions more concrete, we take an example of a pilot research study that was done at a museum exhibit that adopted a mobile eye-tracker to study engagement and climate change learning. Climate change is now considered one of the biggest threats to humans that is worsening with anthropogenic activities and impacting everyday life (e.g., IPCC, 2023; Lohani et al., 2025a). Communicating the irreversible impacts of climate change and engaging community members to act in an environmentally friendly manner is more important than ever (Lohani et al., 2025). Exhibits are an ideal space to spread scientifically informed current challenges and potential solutions to the public (Hamilton and Christian Ronning, 2020; Newell, 2020). The welcoming environment of an exhibit can be helpful in questioning existing beliefs and building an informed understanding of climate change mitigation and adaptation (Janney et al., 2025; Lohani et al., 2025b). Additionally, exhibits are often places of joyful learning among friends and loved ones (e.g., Ash, 2003; Packer and Bond, 2010), thereby making them excellent contexts for grappling with difficult issues.

While designing exhibits, the eye-tracking methodology can be quite helpful in the exhibit development process, as it can help to identify content that matches the goals of the exhibit. Taking the example of climate change, eye tracking can be useful in examining and assimilating content that is thought-provoking and interesting to visitors. While there is limited published work that has utilized mobile tracking to study visual engagement with climate change information, some preliminary work in stationary settings has found support for the use of eye trackers in studying climate change information engagement (Gulhan et al., 2025). In recent work (Oler et al., in press, we examined how attention to information was associated with climate beliefs and attitudes. More detailed information can be gained by studying specific portions of an exhibit, also called areas of interest (AOI). We first describe some technical eye tracking information that can be extracted from an exhibit, followed by its relevance to exhibit designers and researchers.

To display the information that can be gained from a mobile eye tracker, we present data from community members visiting a natural history museum. All protocols followed for this manuscript were approved by the Institutional Review Board at the University of Utah. Before data collection, participants signed the written consent form after the informed consent procedures were followed. Data were collected from 50 museum visitors (*M*_age_ = 36, *SD* = 16). Of these, 40% were Female, 56% were Male, and 4% Genderqueer. Participants were 76% White, 12% Hispanic, 4% Black, 4% Asian, 4% Mixed Race. All participants provided written consent before participating in the study. These data were collected using the Tobii Pro Glasses 3 (2020) mobile eye tracker and software. After data collection, data visualizations could be conducted via custom software (Tobii Pro Lab, 2020).

Before starting data collection, participants were set up with a mobile eye tracker and calibrated. Participants were not provided with specific instructions on what to engage with as they moved through the exhibit. Instead, they were prompted to interact with the exhibit as they typically would and view what they would like to naturally—often referred to as a free-view task. Given the critical information presented in this climate change exhibit, it is extremely relevant for the exhibit designers to know if the visitors are paying attention to and learn from it. An analysis of pilot eye tracking data is presented to showcase how eye tracking data could be used to objectively examine visual engagement with core portions of the museum exhibit through visualization and quantitative eye tracking metrics. For illustrative purposes, in our analysis, we selected a specific portion of the exhibit that provides critical information to visitors about extreme heat-related changes that are linked to climate change; see Figures 3, 4.

## 4 Anticipated results

### 4.1 Benefits of mobile eye-tracking?

Next, we identify several benefits of this methodology. The utilization of eye tracking methodology can be extremely beneficial across a wealth of different contexts. In particular, we highlight several benefits of mobile eye-tracking in the context of exhibits, where it remains underutilized.

#### 4.1.1 Mobile eye-tracking is more ecological and user-friendly than stationary eye-tracking

Mobile eye tracking technology, as compared to stationary eye tracking, has its own advantages because it allows for a much more naturalistic and objective observation of the viewers' experience. While stationary eye-tracking can provide a wealth of information that is applicable to exhibits, it still lacks compatibility with the true experience of visitors. Advanced mobile eye-tracking technology has been shown to be just as accurate when participants are dynamic (e.g., walking) as when they are stationary (Onkhar et al., 2024); however, research has also shown that a chinrest should not be used with a mobile eye tracker as it can adversely impact accuracy (Onkhar et al., 2024). Furthermore, the recent mobile trackers have better accuracy (e.g., Tobii Glasses 3 was better than the previous model due to their advanced illuminators and camera placement; Onkhar et al., 2024) for conditions like walking, which is relevant for museum settings. It can also be easily used with minimal training, making it accessible to anyone interested in adopting it in exhibit work (Bulling and Wedel, 2019). Eye tracking software has also improved substantially in recent years and has now made it much easier for users to process collected data, i.e., quickly extract usable and interpretable information (Bulling and Wedel, 2019).

#### 4.1.2 Real-time and objective assessment of engagement in an exhibit

A key draw of eye tracking is that it provides a way of capturing visual attention in real-time to content of interest, such as text panels, images, videos, and interactive panels. Until recently, the primary way of assessing engagement was through observations, testimonials, interviews, and questionnaires (Museums Association, 2017). With the advances in technology, mobile eye-tracking allows for real-time measurement of continuous engagement while visitors experience and exhibit, which provides a powerful way of assessing engagement and interest without utilizing self-reports from visitors (which can have limitations). Additionally, one of the key benefits of mobile eye tracking is being able to provide within-person analysis by seeing how individuals' chosen behaviors and interactions with environments change their gaze patterns (Fu et al., 2024). Several mobile eye-tracking metrics provide objective measures of visual engagement with an exhibit in real-time, enabling researchers and gallery designers to have the information they need to make decisions about specific items in the exhibit. We utilize a climate change exhibit to elaborate on this point further.

### 4.2 Technical information on mobile eye tracking

Once data collection is complete in real-time, the data are integrated for further processing. In order to access usable information, the raw gaze data of participants needs to be transformed into mapped data. The process of mapping involves imposing raw gaze data over snapshots of the studied area. Broadly speaking, the visual engagement data collected from the visitor (i.e., the gaze data reflecting where the visitor was looking) can be merged with all that the visitor had visually available to them (i.e., the scene video from the visitor's vantage point). This *mapping* allows one to qualitatively and quantitatively analyze visual engagement with an exhibit. Historically, the mapping process has been done completely manually. However, new developments have allowed a large portion of the process to be automated (Bulling and Wedel, 2019).

#### 4.2.1 Data visualizations using mobile eye-tracking: what kind of qualitative indices can be extracted to study exhibits?

**Figure 5:**
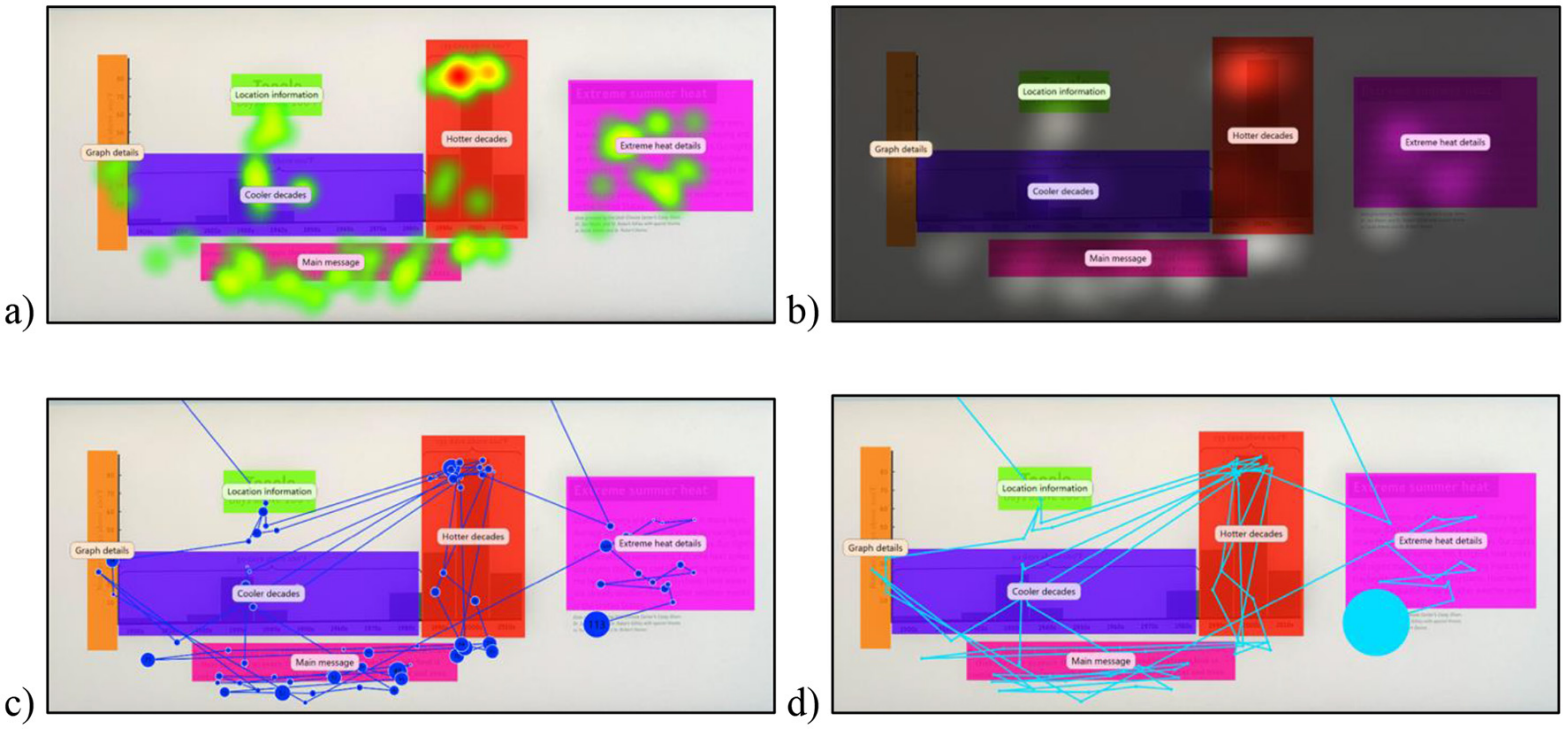
Using the created Areas of Interest, results are presented for the same area of the exhibit, utilizing different data visualizations based on data from one participant. The same portion of the exhibit is presented with. We present four different types of visualizations: **(a)** Heat maps, **(b)** Opacity maps, **(c)** Scan path, and **(d)** Bee Swarm.

After these data are mapped to the exhibit environment, they can be used to create different visualizations. *Visualizations* are a practical way to qualitatively learn about visual engagement patterns. These are some of the most beneficial ways to communicate results as they allow viewers to visually understand the underlying trends (Bojko, 2009). In general, most software accompanying a mobile eye tracker can typically create four common visualization types: heat maps, opacity maps, scan paths, and bee swarms. These visualizations can be created for a group of exhibit visitors (as done in Figure 3 using pilot data from 50 visitors) or for an individual visitor at a time to see their engagement with relevant portions of an exhibit (as presented in Figure 5). The heatmaps (Figure 3a) and opacity maps (Figure 3b) both provide information on where the majority of gaze has fallen, i.e., visualizations capturing the density of visual attention. The *heatmap* visualization provides information through a gradient of colors overlaid on the exhibit image. The regions that attracted the most attention are overlaid with colors to indicate the degree of visualization, and typically, the warmer colors mean the highest level. Inversely, the *opacity map* visualization blacks out most of the image, and the areas with the most gaze are the most visible. The scan path (Figure 3c) and bee swarm (Figure 3d) visualizations provide information on gaze progression in chronological order, i.e., visualizations capturing the timeline of visual engagement. The *scan path* visualization takes each point where the gaze rests and adds numbers indicating order. The *beeswarm* visualization type depicts each individual movement of the gaze chronologically. Bee swarms are usually presented as animations and are most applicable to demonstrate temporal behavior (Blaschek et al., 2017). These are best viewed in video format, as they display the chronological sequence of engagement across portions of an exhibit.

Each type of visualization has its own benefits and is appropriate for different situations. Within the museum context, density visualizations using heatmaps and opacity maps (Figures 3a, b) are best used to convey key areas of engagement of visitors. This allows exhibit designers, curators, and researchers to be able to immediately gather information about what is and is not interesting for visitors. Additionally, heatmap and opacity map visualizations can be created using different metrics; the primary types are fixation count, absolute gaze duration, relative gaze duration, and participant percentage maps (Bojko, 2009). For example, a museum researcher might choose to use a participant percentage heat map when communicating how many visitors will notice a key piece of information that they suspect to be underutilized. This allows for easy and intuitive communication of the areas that are most often viewed. Opacity maps and heatmaps are able to both communicate the same data metrics, however differ in their presentation. Opacity maps can more clearly demonstrate the areas of key relevance to viewers (Špakov and Miniotas, 2007). However, they may not be suitable for viewers unacquainted with the stimulus image, as the darkening effect can prevent first-time viewers from understanding the content of the stimulus. Whereas, heatmaps are able to show a more total view of visual behavior.

As seen in Figures 3a, b, *both* the heatmap and opacity map display that the majority of gaze lands on the two peaks of the graph and the text in the snapshot. Both these visualizations work well with data from a large number of people to get a sense of regions of the exhibit that received the most visual attention, and if the critical regions of the exhibit received the engagement the exhibitors would like, as this would help design effective exhibits. These visualizations can also help identify areas that are overlooked to help the designers improve engagement in line with their goals for the exhibit. Similarly, researchers could also gain an understanding of regions that museum visitors attend and engage with. However, if the interest is not in regions of engagement and rather in the temporal aspects of engagement, heatmaps and opacity maps are not relevant.

On the other hand, the scan path and beeswarm (Figures 3c, d) are timeline visualizations best used to understand what the visitors are drawn to and in what order, specifically to understand where they are drawn first in a scene. This allows curators and exhibit designers to determine if what was intended to be seen first is actually seen first. This is most important for the design of a linear exhibit when the order of visual engagement is critical. Individual scan paths and bee swarm visualizations can be created for each visitor to examine the order in which visitors interact with the whole exhibit. Typically, the scan path and beeswarm visualizations are best when a handful of participants' data are utilized. This is obvious by comparing the visualizations in Figures 3c, d created with data from 50 participants vs. Figures 5c, d that has data from only one participant. The latter is more interpretable and useful to understand the temporal path that a museum visitor took and the areas they engaged with and for how long. Therefore, the scan path and beeswarm visualizations are helpful in pilot testing how museum visitors interact with exhibits and in what order, which can be incredibly useful to make decisions about exhibit design and understanding visual behaviors. However, beeswarms are often not suitable for portrayal as static graphics because they lack the numbering system used in scan paths. They also lack the scan path's ability to demonstrate fixation duration (represented by the size of each dot). However, a key weakness of scan paths and bee swarms is that they are highly susceptible to becoming overwhelming and cluttered when overlaying data from multiple sources. Notably, to avoid creating misleading visualizations, it is best practice to include the fixation parameters (e.g., the number of milliseconds and the degrees of the visual field) and the conditions that generated the visualizations (e.g., heatmap color scale, metric depicted, and viewer task; Bojko, 2009).

#### 4.2.2 Eye tracking metrics: what kind of quantitative indices can be extracted?

Visualizations provide an informative qualitative approach and should be complemented with quantitative analyses. One of the most common metrics is *fixation*, which is a time period when the eyes' gaze is relatively still within an area and stays there for a predefined minimum duration, usually representing intake of visual information and engagement (Rayner, 2009; Russo, 2019). The area and minimum duration to count as a fixation are predefined before processing data; for a detailed consideration (see Hooge et al., 2025; Kümmerer and Bethge, 2023; Startsev and Zemblys, 2023). Existing eye tracking processing software from reputable companies and scholars typically provides the most common algorithms for detecting eye movements, fixations, and saccades, which is a good starting point. However, any processing choices should be made after considering the existing literature and all efforts to make this process transparent and replicable are encouraged (Kümmerer and Bethge, 2023). Once the fixations are identified, follow-up metrics like those listed in Table 1 can be extracted using the software (Bylinskii et al., 2017). One of the most common fixation metrics is the *fixation count*, which is how many times the visitor fixated on a particular area of interest (i.e., the number of fixations). Another important metric is the *fixation durations*, which is a measure of the time period of visual engagement. In addition, it can be useful to examine which areas attracted a visitor's attention, and this information can be provided by the *time to first fixation* (in seconds), which captures how long it takes for the visitor to look at each area of interest. With respect to the museums, the number and duration of fixations provide a way of objectively evaluating engagement with relevant areas of the exhibit, often connected with the importance of an area. Thus, an exhibit designer, curator, or researcher can examine the critical areas of interest within an exhibit and evaluate how visitors are paying attention to them.

**Table 1:** Common quantitative metrics are presented for a single participant.

**Areas of interest**	**Eye tracking metrics**		
	**Total fixation duration (seconds)**	**Time to first fixation (seconds)**	**Fixation count (number)**
Cooler decades	1.38	4.09	7
Extreme heat details	3.21	14.39	8
Graph details	0.68	17.19	2
Hotter decades	4.53	4.75	9
Location information	0.44	5.46	1
Main message	3.42	3.74	10

Another relevant metric for exhibit purposes is saccade. *Saccades* are swift eye movements between fixations and are often associated with search patterns (Nuthmann and Canas-Bajo, 2022; Phillips and Edelman, 2008; Rayner, 2009). The duration of the saccade varies by the goals at hand. For example, scene perception has a longer saccade duration than reading (Rayner, 2009). Saccades duration and length have been utilized to study cognitive processes such as fatigue (Di Stasi et al., 2014) and memory performance (Ohl et al., 2024). In the context of exhibits, saccades can be helpful in learning about how visitors scan an area of the exhibit (e.g., what they look at first and what order they scan different aspects of the exhibit). Similar to visualizations, it can provide useful ways of understanding visitors' interest and behavior. Thus, these metrics can be easily adapted to exhibit contexts as they have been utilized in studying user experience and usability behavior (e.g., Novák et al., 2023; Shen et al., 2020). A commonly adopted method for identifying eye fixations and saccades is the Velocity-Threshold Identification (I-VT) method Salvucci and Goldberg, (2000), which is utilized commonly by custom software, including Tobii Pro Glasses 3 (2020). This method compares the velocity of eye movement to pre-defined time and area thresholds to categorize saccades and fixations (Salvucci and Goldberg, 2000).

Distinctions have also been made between direct and indirect operationalization's that can be made through eye movement data (Hooge et al., 2025). *Direct operationalization* involves accurately measuring eye movements, such as categorizing fixations and saccades, which are possible through research-grade eye tracking hardware and software and implementation of best practices recommended by experts in the field (Hooge et al., 2025; Kümmerer and Bethge, 2023). *Indirect operationalization* is more complicated as it includes utilizing eye movements (e.g., fixation location and duration and saccades) as proxies for psychological processes (e.g., perception, attention, and learning). When making such indirect interpretations, the outcome depends on the definition of operationalization that is chosen by the researcher (Hessels et al., 2024; Hooge et al., 2025) and is contingent upon the environment, thus requiring caution (Mayr et al., 2009). For instance, fixations are most often associated with learning processes and engagement; some ambiguity can remain while making these indirect interpretations (Henderson, 2003; Hessels et al., 2025; Hooge et al., 2025; Negi and Mitra, 2020). As in some contexts, fixation count has been shown to be significantly related to the level of confusion (Salminen et al., 2018).

The difficulty of interpreting eye movements remains a continued issue within the field of eye-tracking research today (for details, see Hessels et al., 2025; Hooge et al., 2025). However, a thoughtful research process can be quite useful in making careful interpretations. For instance, there are opposing effects of mental workload that are due to perceptual load (that can lead to shorter fixation durations and more frequent fixations) vs. cognitive load (that can lead to longer fixation durations and less frequent fixations; Liu et al., 2022), and can help make interpretations about how visitors may experience workload at different stages of processing. Furthermore, recent reviews of existing literature on utilizing eye tracking methodology in the context of education to make interpretations about cognitive processes (Liu and Cui, 2025) are also a helpful resource for newer studies to learn about relevant findings to make informed indirect operationalizations. Improvements in the interpretation of cognitive processes tied to mobile eye tracking remain an important direction for applied mobile eye tracking research.

### 4.3 Challenges in adopting mobile tracking for exhibit research and development

#### 4.3.1 Limitations to internal validity

Above, we discussed limitations around the interpretability of eye tracking metrics. Transparency in sharing decisions made for computing eye-tracking parameters in every study remains essential as this will promote comparison of findings across studies (Hooge et al., 2025). While research in museum exhibits via eye tracking methodology is limited, much foundational work can get established with additional research. Even though one advantage of ambulatory eye-tracking studies is that they are naturalistic and provide high external validity, considerations of proper internal validity are necessary as well. For instance, while some eye trackers maintain stable gaze estimates despite slippage, jostling, or bumping during movement, others experience a decline in accuracy under these high activity conditions (Niehorster et al., 2020). It is important to consider the data quality metrics reports shared by the mobile eye tracker company to get a sense of their accuracy (e.g., Tobii AB, n.d.). Similarly, the accuracy of automated mapping of gaze data can be inaccurate and ineffective, especially with dynamic and open environments (Hahn and Klein, 2023) present in museum exhibits. Even though automatic mapping can help reduce manual mapping, that is time-consuming and effortful, it is worth noting that a manual examination is still necessary for quality control and minimal inaccuracies. Notably, mobile eye-trackers may be unsuitable for studies involving small AOIs or very few fixations, as the automatic gaze mapping performed by mobile glasses software often lacks the precision needed (Hahn and Klein, 2023). For instance, they may be unable to detect word-level differences in fixation duration. Additionally, small changes in environmental stimuli can have large implications on visual attention and behavior (Kiefer et al., 2017), but it can be nearly impossible to control every aspect of the environment in an exhibit setting. Other visitors, noise level, and exhibit busyness can play a role in how a person chooses to interact with an exhibit, but can be difficult to control, especially when the interest is in naturalistic museum settings. If possible, perhaps less busy times of the day could be targeted for data collection. Researchers should be encouraged to manage such environmental variables when possible, but to otherwise accept this limitation in order to allow for the naturalistic benefits of eye-tracking.

#### 4.3.2 Physical barriers to eye-tracking data collection

When designing ambulatory eye-tracking studies, it is crucial to consider several limitations. First, mobile eye-tracking devices are designed to be worn by individuals with normal vision and without any obstructions to their eyes. Participants who wear corrective lenses are often excluded from eye-tracking studies because eyewear lenses reflect the infrared light used for measuring eye position, potentially interfering with the accurate tracking of the pupil (Carter and Luke, 2020; Stuart et al., 2016). However, some mobile eye-trackers have recently introduced attachable lenses (sold for an additional cost) that correct for common forms of near and farsightedness (Tobii AB, 2020). However, for those museum visitors with complex prescriptions, this limitation may currently still exist as the attachable lenses cover generic prescriptions only. For similar reasons, drooping eyelids, long eyelashes, dark makeup, or cataracts can also result in increased eye-tracking errors (Carter and Luke, 2020; Holmqvist et al., 2011). Since older adults are more likely to wear corrective glasses or to have other eye abnormalities, excluding people according to these criteria could compromise the randomness of the sample imposing significant barriers to research focused on older adults, and it remains an important consideration for researchers.

Another barrier that requires consideration is the ethical challenge at play when a person is wearing an ambulatory eye tracker in a public space. Appropriate information should be shared with those who are not a part of the study for them to decide if they prefer not to be around an eye tracker while it is recording data. The requirements set by the institutional review board will dictate these conditions too. Researchers could collect data in off-hours when others are not around to bypass this issue as well. However, additional considerations would be needed if the visitor wearing the eye tracker may have to interact with other visitors who are not wearing an eye tracker or are not involved in the research (Mayr et al., 2009).

An additional consideration for utilizing mobile eye trackers is the environment. Mobile eye tracking's greatest strength is its ability to allow researchers to investigate non-laboratory environments and produce data in applied settings. However, some environments present additional struggles because of light and overheating. The issues with light are twofold. Firstly, with significant levels of glare, participants will begin to squint, lowering data quality (Armato et al., 2013). Additionally, sunlight can lower data quality because of competing infrared light, worsening the efficacy of the embedded illuminators (Imabuchi et al., 2016). However, some new generation eye trackers are more prepared to deal with this challenge. For example, the Tobii Pro Glasses 3 can be outfitted with protective lenses that allow for protection from glare. Work is also currently being done to improve the ability for eye trackers to be used outdoors in high-light conditions (Rusnak et al., 2025). Though current generation eye trackers have approved operating temperatures up to 113°F/45 °C, at high temperatures, overheating becomes more common, especially when exposed to direct sunlight (Tobii manual, 2020).

#### 4.3.3 Mobile eye-tracker cost

In the current mobile eye-tracking landscape, there is a trade-off between cost and user-friendliness. The easiest-to-use eye trackers provide software that transforms enormous data files of pupil position and cornea reflection into interpretable gaze paths and fixation points, even providing helpful visualizations. However, these eye trackers can be very expensive. On the other hand, less expensive options require the researcher to analyze and interpret the raw data themselves, which requires advanced computer coding skills to create a process that is automated by custom software. However, with some consideration, it will be possible to use existing custom software with cheaper eye trackers as well. In fact, some open-source software are already available (Nasrabadi and Alonso, 2022). At the same time, with advancing technology, ongoing and future work may make ambulatory eye-tracking more cost-effective by using participants' smartphones (Gunawardena et al., 2024; Valliappan et al., 2020). Additional guidelines on tools available to conduct eye tracking are also available (see Niehorster et al., 2025).

## 5 Discussion

### 5.1 Tips and tricks to consider while adopting mobile eye-tracking of exhibits

#### 5.1.1 Practical recommendations

While determining the mobile eye tracker, the nature of the tasks participants would be doing would matter. If the dynamic nature of the task includes standing and walking, the mobile trackers are about to accurately measure visual gaze; however, with tasks like skipping and jumping, the accuracy can drop (Hooge et al., 2023). Mobile eye-trackers like Tobii Glasses 3 have been found to be suitable for walking conditions, but the accuracy can drop for high movement conditions such as jumping and skipping (Hooge et al., 2023). Experimenters are encouraged to adopt eye trackers with a higher sampling rate if higher movement is expected, as it is found to handle fast movement better (Hooge et al., 2023).

Despite the busy environment of an exhibit, planning ahead on how to use mobile tracking can make its adoption highly feasible. While the data collection will happen in the exhibits section, the setup can be done in a separate room. If possible, finding a less noisy room can be helpful. The team conducting the study may benefit from having a checklist for the before, during, and after steps for getting all done seamlessly. Before data collection, it is important to have the mobile tracker and laptop fully charged. If informed consent is needed (depending on the study and institution), the required procedures can be completed before starting the setup. Depending on the eye-tracker, having a breathable bag to keep the small recording unit (as shown in Figure 1) is helpful. Also, it helps to inform the participant of certain behaviors that may disrupt data collection quality (e.g., refrain from placing fingers on the lens of the eye tracker).

Before the data collection starts, it is important to calibrate the trackers (which allow the tracker to learn where the person is looking). After calibration, it is also a good practice to make sure that the tracker is actually doing a good job tracking the participants' eyes by asking them to look at exact locations and checking on the software that the real-time recording is accurate. Such validations can be very helpful in collecting accurate and valid data. Also, it is helpful to have a reminder to hit record (often, that is another step); otherwise, offline data won't be available. Any other instructions (where to go in the exhibit, etc.) can also be shared before starting the data collection because it is best to make it as least disruptive as possible. Before starting with the main exhibit region, it is helpful to have some “practice” time built in so that participants get used to the setup and are more naturally interacting with the exhibits of interest.

The researcher should also consider their interest and need to be able to monitor the live feed generated by the mobile eye-tracker. Generally, the recording does continue even when the live feed may be discontinued due to the eye tracker being out of range because the recording unit is with the participant along with the eye tracker. The Wireless Local Area Network (WLAN) will limit the maximum distance the researcher can view the live feed on the computer receiving the wireless signals from the mobile tracker. The maximum distance to be able to receive the live feed is dependent on the environment and is informed by factors such as high radio traffic. The range is more outside than indoors. Additionally, the live view can reconnect when the eye tracker comes back into range allowing researchers to stop recordings when data collection is finished.

After eye tracking is complete, the participants could be brought back to a quiet space where the setup was initiated for post-exhibit data collection (e.g., interviews and surveys) and debriefing purposes. Improving the protocol with multiple pilot participants before collecting the main data is also quite useful. As expected, all used products should be cleaned before being used by another participant. Additional technical information is available for adopting eye-tracking in research and analysis (for more information, see Carter and Luke, 2020; Hessels et al., 2025; Kasneci et al., 2024; Rayner, 2009).

#### 5.1.2 Calibration tips and troubleshooting

The eye-tracker calibration process can present additional limitations. Mobile eye trackers require calibration each time a new wearer uses them to ensure accurate measurements. This calibration process aligns eye movements with the visual field, enabling the device to map gaze to perceived objects (Slone et al., 2018). While this process is normally straightforward, it is helpful to know how to execute the calibration process properly (Nyström et al., 2013). In general, calibration errors can be reduced by presenting the calibration target at the participant's eye level and making sure they are at a proper distance away from the calibration target. If using eye-tracking software, it can sometimes be helpful to restart the software prior to calibration if multiple error messages occur. This is because after sitting idle, the communication between the glasses and the laptop unit can be disrupted, and restarting the system fixes this issue by re-establishing the connection. Furthermore, additional suggestions are available to collect high-quality eye-tracking data by paying attention to factors such as corrected vision, eye characteristics, and makeup (Nyström et al., 2013). Another recommendation is to conduct re-calibration, especially when the study is long or participants are constantly moving around (Shen et al., 2020). This can be done in between sessions by asking participants to look at specific locations to verify that the eye tracker is accurately detecting the gaze of the wearer in real time.

#### 5.1.3 Challenges in eye-tracking analysis

It is also helpful to consider data analysis plans before starting the main data collection. Significant progress has been made in properly analyzing eye-tracking data, and custom software is available to assist in this process. The visualization features provide rich information to exhibit curators, designers, and researchers to understand how visitors interact with the exhibits. Offline indices can also be created, but they may need the support of a research collaborator familiar with these additional features. However, most eye-tracking companies have been providing additional resources to make this viable.

At the same time, certain aspects of the analytical process still need further consideration. A primary consideration is which areas of the exhibit are of most interest for the project at hand. In order to maximize generalizability, a two-step process is effective. First, the team should determine which areas in the exhibit are of interest to examine, i.e., the AOIs. These are informed by the rationale for collecting data and which regions matter the most. Second, as a follow-up process, feedback can be collected from others to evaluate if the chosen areas meet the needs or if additional regions should be edited. If there is room to make this process more objective, ratings can be collected for each of the regions on the dimensions of interest, such as relevance or interest. While designing the areas of interest, the software often allows one to load the same template across participants (so that variability in drawing them does not add noise). Failing to follow best practices for the creation of AOIs could lead to misinterpretation of each AOI (Orquin et al., 2016; Hooge et al., 2025). It is a good practice to conduct frequent quality checks using scan path visualizations to look for issues such as missing data (suspiciously few scan paths; Holmqvist et al., 2011). Finally, pilot studies are an effective way to identify many data collection and analysis issues and are highly encouraged (Hessels et al., 2025).

### 5.2 Concluding remarks

Mobile eye-tracking is a state-of-the-art, science-based technology that provides objective, real-time information on how visitors can learn by exploring learning exhibits. The methodological and applied information covered in this paper can be adapted by exhibit curators, designers, and researchers for their improving engagement and learning at exhibits. The eye-tracking technology can help advance the mission of museums as public institutions by creating engaging, informative, and meaningful exhibits for their communities. It also exemplifies the truly interdisciplinary effort that is required to create welcoming and informal spaces through museum exhibits for learning and growth. We hope that this work sparks an interest in mobile eye tracking as a tool to advance exhibit engagement further.

## Data Availability

The original contributions presented in the study are included in the article/supplementary material, further inquiries can be directed to the corresponding author.
